# Supplementary material for: Ocular-following responses in school-age children
Source: PLoS One. 2022 Nov 10;17(11):e0277443. doi: 10.1371/journal.pone.0277443 (PMC9648761; doi:10.1371/journal.pone.0277443)
Supplement: Attachment — Submitted filename: Notes to Authors.pdf [file pone.0277443.s001.pdf]

Notes to Authors

1. Please see my notes throughout regarding concerns for the timing of the framing intervals (80ms, 160ms) to measure the OFRs in children.

4. The written English is adequate with the exception of noted corrections in my line item notes below.

5. Please see specific notes below.

Abstract

Line 22: Recently, it has been…

Line 23: and, thus, might have…

Lines 31-32: … recording sessions. Although, we found… (And, welcome to working with kids – A LOT of variability! ;))

Introduction

Line 48: humans and *non-human* primates…

(Line 62: random thought – also, maybe, non-verbal school-aged kids and adults? Added to the discussion?)

Line 66: write out these numbers?

Lines 70-81: These lines seem to read more like an abstract than a general purpose statement to setup the focus of the study/paper. As for the purpose: to be the first to search for/record OFR's in children, to determine if/when these movements are developed,…?

Lines 70-72: *Returning after reading the methods…* Revisiting the whole of this work, it seems to have dual purpose: (1) to develop a custom, video eye-tracking device for use with children in a clinical setting, as well as to verify its validity compared to a gold standard such as a search coil; and (2) attempt to measure and document OFRs in children aged 6-14. The first of these may be its own paper? Maybe not, yet it seems to have led to an addition of introductory information and results mixed into the Methods section.

Methods

Line 84: "*Sixteen* healthy…"; please write out this number, especially being at the start of the section.

Lines 89-90: What was the timing between the complete exam and the research visit? The same say was not likely if they were dilated during the exam? If returning for a second visit, how much time had past? This could be important if subjects where prescribed a new/updated spectacle prescription and needing an adaption period to the new glasses.

Line 96: A reference to the setup shown in Figure 1a?

Line 102: "(*Figure* 1b)."

Line 109: "… composed of *four randomly-intermixed* trials…"

Lines 117-122: I like this addition! ☺

Lines 123-181: This seems to be a separate, but related!, study. Please refer to my comments for the purpose statement at the end of the Introduction. The Hopf paper from 2018 (doi: 10.1371/journal.pone.0204008) might be of assistance in terms of the organization/format for what I am recommending. It seems distracting to have additional information introduced and results reported within the Methods section.

Line 144: "…(*Figure* 1a)."

Lines 144-146: I can see the authors' argument for the three frames of data collection, vs continuous data, yet is using 80 ms for the latency appropriate for use with younger children? Other eye movement studies, including the Hopf paper mentioned above, have shown that most eye movement dynamics are adult-like with the exception of latency periods decreasing into the pre-teen/teenage years. Therefore, would half of your study population have a longer latency period and, then, require a different framing rate to measure position/displacement?

Lines 168-171: I appreciate that the authors calibrated the system to a standard measure to validate their protocol. (Again, maybe a separate purpose/paper?) Were both the two subjects adults? Authors? If so, could this be done with an older child? Many children are very successful with contact lens wear so finding one or two 10-12-year-olds to wear a search coil may be possible? (All, of course, pending IRB approval.)

Line 177-178: If this is true, you have the data to do so? An argument to first publish this as a smaller paper first vs the dual purpose option?

Lines 194-197: Looking at the Hellstrom paper (1997 vs 2009?), this is an axial length measure? Wouldn't AL be easy to measure on your subjects to verify an average used? As mentioned, a small difference would not matter, yet does your population fits this report from the literature, especially if comparing a 6yo with a 13 yo? Italians vs Swedes? I would predict that you would have more variability with your older subjects given the increase in the prevalence of myopia over the past 25 years. If the author's calculations can show that a range of ALs do not significantly change the degrees/pixel, the average used is reasonable. However, the reference provided seems dated when there is a TON of current literature on axial length in school-aged myopes, including controls.

Line 202: The head was stabilized (Line 92) during testing so large head movements should have been limited?

Line 209: "… the *middle* 68th percentile of *these* values,…"

Lines 209-210: I like the use of the mid-68th percentiles. Maybe a table to show the amount (number or percent) of trials dropped for each subject? Or, a column for reliability added to Tables 1 & 2?

Lines 210-212: This belongs in the results section?

Lines 245-254: This section is typically listed at the beginning of a methods section? Also, what about the two subjects used to calibrate/verify the eye tracking device? Or, were these two authors?

Results

Line 258: “In *Figure* 2,…”

Line 260: “(*S*ubject 10).”

Lines 260-262: Please see my previous concern regarding the use of these timing frames.

Line 270: “Note*, however,* the large…”

Line 277: “In *Figure 3,* scatter plots…”

Line 293: On the top row of Table 1, the third and fourth columns should be rightward and leftward?

Line 295: Change in x? Horizontal eye displacement?

Lines 300-315: Some of this seems to include a discussion of the results and should be moved. Please make a simple statement of the results here.

Lines 300-304: Would it first be beneficial to runs trials with continuous measures to plot the instantaneous velocities for children and estimate their latencies as a function of age? (Please see my previous notes on latencies of eye movement dynamics in children, Lines 144-146. This could potentially be another goal/measure to add to the study or another separate paper?) If the latency is different from that of adults, at least for the younger children, the, would you need to use different time intervals? The quantification based on use of time displacements seems an adequate method for OFR measures provided that these latencies are appropriate.

Line 315: “…this variability…”; may need to define if moving/updating preceding paragraphs.

Line 339: “intact” vs “fully developed?” More on this in my overall comments.

Discussion

Lines 347-349: Yes – agreed!

Lines 349-351: Something for future study, I do think that maintaining fixation in infants will be easier than you think; please see literature on Teller Acuity.

Lines 352-354: Again, the OFR is “intact” by age six years. I would like to see further discussion of how this might compare to other types of eye movements and the characteristics in children, especially as most dynamics are adult-like. (The exception is with latency.)

Lines 354-362: I like where the authors are leading to here. I’d like to see this more developed into further discussion. Other eye movements (fixations, saccades, pursuits, vergence) are seen in infancy. Then, specifically, how can this development of OFR’s be used to diagnose other developmental issues?

Line 361: "… important *because, obviously,* the ocular…"

Lines 363-364: "… induce robust *OFRs in adults [13]."* Also, this first sentence of the paragraph seems to be displaced.

Line 369: "15 to 20," or "15 out of 20?"

Line 370: "… smaller *would, however, require* either…"

Lines 374-377: Good! Maybe consider verifying with an older child? (Many kids are successfully wearing CLs by 10-12 years of age so a search coil for 1-2 subjects would be reasonable? If IRB approved, of course.)

Line 377: "*An angle of* 0.2^o is thus…"

Line 382: "*Forty* years…"

Line 383: "… tool for tracking *visual* development…"

References

Lines 433-5: I looked up this article out of interest and a question on Lines 194-197. Volume 75 was published in 1997, not 2009 (https://pubmed.ncbi.nlm.nih.gov/9374253/). The authors may need to further proofread/verify the references.

General Comments

Overall, I agree that the authors have presented a well-written argument that OFRs are present in children. They have successfully developed and verified an eye tracking device to measure such OFRs. I still seem to be stuck on the latency of OFRs in children compared to adults. Given the short latency of these eye movements, it may not be significant, yet it seems to be a gap in the literature -- especially as other types of eye movements (fixations, saccades, pursuits, vergence) are present in infancy yet have latencies that tend to decrease into puberty. It may be beneficial to try to measure OFR latency in children first. (If different from the 80ms used, the measured displacements will be within different framing measures and inaccurate. And, Gellman (ref #13) and Leigh & Zee report 70ms in adult humans.) The authors likely have appropriate means to be able to do this. This could mean that the study could be further strengthened by proposing a three-part goal:

(1) to develop and verify an eye tracking device for clinical measurements of OFRs in children,
(2) (2) to estimate the latencies of of OFRs in children, and then,
(3) (3) to measure horizontal and vertical displacements of OFRs in children, comparing them to those of adults.

These final measurements could then be used for detection of other visual developmental delays such as strabismus and amblyopia.

Articles on eye movement dynamics with age, including latency periods

(just a start, but what I found in less than a few minutes)

Quan, 2002 (PMID: 12202513)

Sinno, 2020 (DOI https://doi.org/10.3766/jaaa.19049)

Irving, 2006 doi:https://doi.org/10.1167/iovs.05-1311)

Salman, 2006 (https://doi.org/10.1016/j.visres.2005.06.011)
