# Supplementary material for: Ocular-following responses in school-age children
Source: PLoS One. 2022 Nov 10;17(11):e0277443. doi: 10.1371/journal.pone.0277443 (PMC9648761; doi:10.1371/journal.pone.0277443)
Supplement: Attachment — Submitted filename: Repsonse to authors (OFRs in Kids).pdf [file pone.0277443.s003.pdf]

I thank the authors for their updated versions and explanations of the many comments. They have shown that OFRs are present in young children using their eye tracking device. I have just a few things to add.

Introduction
I appreciate the expanded explanations in the introduction. It reads more smoothly and provides a clearer purpose statement.

Methods
I would have liked to seen a little more inclusion criteria included with the study population. In the previous review, I asked about the timing of the study visit with the patient exam and potentially prescribed spectacle changes. The authors provided a good explanation yet this should be included in the text of the paper. The later comment about axial length and lack of significant myopia could also be included here.

I agree with the authors' argument for the three frames of data collection and never saw the latency issue as a fatal flaw, just as a gap in current knowledge. However, I wanted a better explanation why that particular interval was chosen. I feel the authors have addressed this in the Discussion.

The flow of the methods still seems a bit choppy with the detour about the calibration of their device and protocol as it is lengthy and distracts from the original purpose. The authors made a comment expecting readers to skip over a lot of the methods, and this distraction may encourage this. Adding an appendix to the paper may be a more appropriate way to include the development of device so that it does not take away from the flow of the paper.

For what was previously line 209, an error still exists, " ...the middle 68th percentile of *these* values, ..."

Results
I like the change in wording to "well developed" for the OFR in children. Perhaps an area of future research can be to measure latencies as well as shorter intervals to determining when the OFR is fully adult like? Much possibility here!

Discussion
I appreciate the authors' succinct additions to the discussion section.

Overall comments
I appreciate the author's attention to concerns raised in the comments from both reviewers. The authors seem well versed in techniques and current literature. They raised many great discussion points from both sets of responses stimulating conversation regarding an explanation of reasoning. Some of the statements could very well be included within the text of the paper itself to clarify the work and make it more accessible to others.

One general concern, I am not sure how much experience the authors have working with children. Some of their comments could be interpreted as rather negative when mentioning working with children. Many kids will surprise you and complete tasks better than some adults. (I know what a search coil is and regularly perform Goldman tonometry on 6 year olds, an exam element many adults cannot successfully withstand.) Having done eye tracking with calibration on preschoolers, it is possible. Infants will readily fixate on flashing screens while their parents can hold heads or in airplane position; we do this at a slit lamp during routine eye exams. Additional testing with devices may potentially lengthen a comprehensive exam for a child, yet this is already being done. In the next few years, I see axial length measures becoming standard protocol for any young myope. Eye care professionals already use VEP on infants and pre- or non-verbal children if concerned about developmental issues. (Not a fan of it, but it is commonly performed.) Anyway, I mentioned this not so much a specific need for this article. The authors have potential for future work based on these findings and should not see young subjects as any more challenging than other populations. Kids are also far more fun!
