# Supplementary material for: Ocular-following responses in school-age children
Source: PLoS One. 2022 Nov 10;17(11):e0277443. doi: 10.1371/journal.pone.0277443 (PMC9648761; doi:10.1371/journal.pone.0277443)
Supplement: Attachment — Submitted filename: ReplyLetter.docx [file pone.0277443.s004.docx]

We thank the editor and reviewer for their suggestions, which we have incorporated. A point-by-point reply to their comments follows.

**Editor**

*The authors have satisfactorily addressed the majority of the comments from the reviewers. Only a couple of minor comments from one of the reviewers remain to be addressed:*

*"I would have liked to [have] seen a little more inclusion criteria included with the study*

*population. In the previous review, I asked about the timing of the study visit with the*

*patient exam and potentially prescribed spectacle changes. The authors provided a*

*good explanation yet this should be included in the text of the paper. The later comment*

*about axial length and lack of significant myopia could also be included here."*

We addressed this comment in full, see below.

*In regard to the reviewer comment about perhaps moving the portion of the methods in which the calibration of the device and protocol is described to an appendix, some readers will likely want to know that this is an important aspect of the paper. Therefore, while moving that material to an appendix does not have to be the approach, please add a sentence to the abstract to indicate that methodological considerations/development of the device is described in the paper.*

We are not too keen on appendices (or supplementary material), unless it is strictly necessary, and we agree with the Editor that some readers might find the methodological aspect important (hence our detailed description of it). We added a sentence in the Abstract and one in the Introduction to indicate that we describe it. The relative section is well labeled, so it will be easy for interested readers to find it, and for uninterested readers to skip it.

**Reviewer #1**

*I would have liked to seen a little more inclusion criteria included with the study population. In the previous review, I asked about the timing of the study visit with the patient exam and potentially prescribed spectacle changes. The authors provided a good explanation yet this should be included in the text of the paper. The later comment about axial length and lack of significant myopia could also be included here.*

We have expanded the description of the inclusion criteria in the Study population section of the methods, along the lines of our previous response. In the Data analysis section we also reiterated that the assumption we made about eye diameter is justified because of the low refractive error in our population.

*The flow of the methods still seems a bit choppy with the detour about the calibration of their device and protocol as it is lengthy and distracts from the original purpose. The authors made a comment expecting readers to skip over a lot of the methods, and this distraction may encourage this. Adding an appendix to the paper may be a more appropriate way to include the development of device so that it does not take away from the flow of the paper.*

We realize that it is quite a lot to get through, but we feel that some readers might find it important. Since it is all contained in its own labeled section, it would be easy for uninterested readers to skip just it and focus on the rest of the methods. We find that appendices are often even more disruptive to the flow, especially when articles are read on digital devices, on which skipping a section is easy, but navigating to the end to find the appendix, and then back to where one was, is often cumbersome. We thus try to avoid them.

*For what was previously line 209, an error still exists, " ...the middle 68th percentile of these values, ..."*

Fixed.

*I appreciate the author's attention to concerns raised in the comments from both reviewers. The authors seem well versed in techniques and current literature. They raised many great discussion points from both sets of responses stimulating conversation regarding an explanation of reasoning. Some of the statements could very well be included within the text of the paper itself to clarify the work and make it more accessible to others.*

We understand the reviewer’s point. We addressed some of the points raised in the previous round only in the reply letter because we tried to strike a compromise between being fully exhaustive and being concise. We have made a good faith effort to include in the manuscript everything that might be of more general interest, while leaving to the reply letter what might be of more special interest. Although we realize that where the line lies is always somewhat subjective, we believe that there is value in trying to keep the discussion as focused as possible.

*One general concern, I am not sure how much experience the authors have working with children. Some of their comments could be interpreted as rather negative when mentioning working with children. Many kids will surprise you and complete tasks better than some adults. (I know what a search coil is and regularly perform Goldman tonometry on 6 year olds, an exam element many adults cannot successfully withstand.) Having done eye tracking with calibration on preschoolers, it is possible. Infants will readily fixate on flashing screens while their parents can hold heads or in airplane position; we do this at a slit lamp during routine eye exams. Additional testing with devices may potentially lengthen a comprehensive exam for a child, yet this is already being done. In the next few years, I see axial length measures becoming standard protocol for any young myope. Eye care professionals already use VEP on infants and pre- or non-verbal children if concerned about developmental issues. (Not a fan of it, but it is commonly performed.) Anyway, I mentioned this not so much a specific need for this article. The authors have potential for future work based on these findings and should not see young subjects as any more challenging than other populations. Kids are also far more fun!*

We have extensive experience in recording eye movements in adults and school-age children, but not much in infants. We have heard mixed stories from colleagues that have, but we find the reviewer’s comments encouraging. We thank the reviewer again for the many insightful comments.
