# Supplementary material for: Ocular-following responses in school-age children
Source: PLoS One. 2022 Nov 10;17(11):e0277443. doi: 10.1371/journal.pone.0277443 (PMC9648761; doi:10.1371/journal.pone.0277443)
Supplement: Attachment — Submitted filename: ReplyLetter.docx [file pone.0277443.s005.docx]

**Editor comment:** Thank you for submitting your manuscript to PLOS ONE and for your thorough responses to the reviewer comments. Only one item remains: Please submit a revised version of the manuscript that addresses this final point regarding the abstract and described below.

Please add the following to the abstract, as the readership will likely be interested in knowing that there are many methodological details in the paper: add a sentence to the abstract to indicate that methodological considerations/development of the device is described in the paper. As the abstract reads now, it is not clear that all of the details in the development of the method are presented in the manuscript.

**Response:** Dear Editor, thank you for your comment. We modified the abstract accordingly and added the sentence regarding the developed custom system used for OFRs recording in our study.

**Reviewer comments:** n/a

--

The authors would like to thank again the Editor the comment, as well as, reviewers for their previous comments that allowed us to improve the paper.
